# Immunohistochemical analysis of Tn antigen expression in colorectal adenocarcinoma and precursor lesions

**DOI:** 10.1186/s12885-022-10376-y

**Published:** 2022-12-07

**Authors:** Gabrielle E. Dombek, Ana Sofia Ore, Jane Cheng, Yasuyuki Matsumoto, Jonathan N. Glickman, Aaron Fleishman, Jamie Heimburg-Molinaro, Vitaliy Y. Poylin, Anne Fabrizio, Thomas Cataldo, Evangelos Messaris, Richard D. Cummings

**Affiliations:** 1 Department of Surgery, Beth Israel Deaconess Medical Center, Harvard Medical School, CLS 11090, 3 Blackfan Circle, Boston, MA 02115 USA; 2 Department of Pathology, Beth Israel Deaconess Medical Center, Harvard Medical School, 330 Brookline Avenue, E106, Boston, MA 02115 USA; 3 Department of Surgery, Beth Israel Deaconess Medical Center, Harvard Medical School, 185 Pilgrim Road, Deaconess 207, Boston, MA 02115 USA; 4 Department of Surgery, Northwestern Medical Group, Feinberg School of Medicine, Arkes Family Pavilion, 676 North St Clair Street, Suite 650, Chicago, IL 60611 USA; 5 Department of Surgery, Beth Israel Deaconess Medical Center, Harvard Medical School, 330 Brookline Ave, Gryzmish 6, Boston, MA 02215 USA; 6 Department of Surgery, Beth Israel Deaconess Medical Center, Harvard Medical School, CLS 11087, 3 Blackfan Circle, Boston, MA 02115 USA

**Keywords:** Tn antigen, STn antigen, Colorectal Cancer, Transitional Margin, Immunohistochemistry, Anti-Tn antibody

## Abstract

**Background:**

The Tn antigen (CD175) is an O-glycan expressed in various types of human adenocarcinomas, including colorectal cancer (CRC), though prior studies have relied heavily upon poorly characterized in-house generated antibodies and lectins. In this study, we explored Tn expression in CRC using ReBaGs6, a well-characterized recombinant murine antibody with high specificity for clustered Tn antigen.

**Methods:**

Using well-defined monoclonal antibodies, expression patterns of Tn and sialylated Tn (STn) antigens were characterized by immunostaining in CRC, in matched peritumoral [transitional margin (TM)] mucosa, and in normal colonic mucosa distant from the tumor, as well as in adenomas. *Vicia villosa* agglutinin lectin was used to detect terminal GalNAc expression. Histo-scoring (H scoring) of staining was carried out, and pairwise comparisons of staining levels between tissue types were performed using paired samples Wilcoxon rank sum tests, with statistical significance set at 0.05.

**Results:**

While minimal intracellular Tn staining was seen in normal mucosa, significantly higher expression was observed in both TM mucosa (*p* < 0.001) and adenocarcinoma (*p* < 0.001). This pattern was reflected to a lesser degree by STn expression in these tissue types. Interestingly, TM mucosa demonstrates a Tn expression level even higher than that of the adenocarcinoma itself (*p* = 0.019). Colorectal adenomas demonstrated greater Tn and STn expression relative to normal mucosa (*p* < 0.001 and *p* = 0.012, respectively).

**Conclusions:**

In summary, CRC is characterized by alterations in Tn/STn antigen expression in neoplastic epithelium as well as peritumoral benign mucosa. Tn/STn antigens are seldom expressed in normal mucosa. This suggests that TM mucosa, in addition to CRC itself, represents a source of glycoproteins rich in Tn that may offer future biomarker targets.

**Supplementary Information:**

The online version contains supplementary material available at 10.1186/s12885-022-10376-y.

## Introduction

Adenocarcinoma of the colorectum (CRC) is the third leading cause of cancer-related death in the United States as well as worldwide [1]. This is due in large part to discovery at advanced stages of disease, with only 39% of patients being diagnosed at a localized disease stage [2]. Histopathologic evaluation of endoscopically sampled colonic lesions has thus come to play a critical role in the accurate and expedient diagnosis of CRC. CRC develops through the progressive transformation of the colonic mucosa from normal to dysplastic precursor lesion and ultimately to malignancy. The morphologic and molecular genetic stages in this progression are increasingly well characterized [3, 4]. Alterations in glycan processing of membrane glycoproteins in CRC have been appreciated for some time [5–7], but the exact sequence of glycan changes, and relationship to early stages in carcinogenesis, are less well understood.

Under physiologic circumstances, O-linked glycosylation is a universal post-translational modification, and abnormalities in O-glycosylation have been described in many carcinomas, including CRC [7, 8]. Among the O-glycan changes associated with CRC is the expression of specific, abnormally truncated glycans [9], such as the Tn antigen (﻿CD175, GalNAcα1-*O*-Ser/Thr) and its sialylated form sialyl Tn (STn) (CD175s, Neu5Acα2-6GalNAcα1-*O*-Ser/Thr). These are tumor-associated carbohydrate antigens (TACAs) ﻿which have been identified in a broad spectrum of adenocarcinomas including CRC [10, 11].

The Tn antigen is a precursor structure biosynthesized in the Golgi apparatus by a family of twenty different polypeptide-N-acetylgalactosaminyltransferases (ppGalNAc-Ts), which transfer GalNAc from the donor UDP-GalNAc to a Ser or Thr residue in glycoproteins. The Tn antigen subsequently serves as the common core to which glycans are extended first by the T-synthase and its private molecular chaperone Cosmc to form ﻿primarily core 1 structures (Galβ1-3GalNAcα1-*O*-Ser/Thr, the T or TF antigen), which are precursors to ﻿branched core 2 structures; other O-glycans in colorectal glycoproteins include ﻿core 3 and core 4 O-glycans [12–14].

In most healthy tissues, the Tn antigen does not accumulate due to its complete conversion to extended O-glycans. In pathologic states including various malignancies, however, increased expression of the Tn and STn antigens has been correlated with tumor progression, metastasis, and poor prognosis [15]. Notably, the conversion of Tn to STn antigen requires sialylation, a relatively infrequent cellular process which occurs to varying degrees in the context of aberrant physiologic states, making Tn antigen the predominant epitope in terms of relative abundance in cells; the Tn antigen in specific, therefore, has been the focus of many prior studies given its potential as a relatively abundant mucin-based target [9, 11].

Patterns of Tn antigen expression in CRC have been assessed historically using GalNAc-binding lectins, such as *Vicia villosa* agglutinin (VVA) and *Helix pomatia* agglutinin (HPA), or with antibodies that are often inadequately characterized for specificity [12]. These approaches have shown elevated but variable Tn expression in CRC, in up to 90% of tumors, with STn antigen expression paralleling that of the Tn antigen [16–18]. However, very few studies have directly compared paired tumor and normal colorectal mucosa from the same individual. Instead, they have used samples from healthy subjects as control tissue and therefore lack internal controls [10].

Prior investigation of Tn expression in paired tumor and normal colorectal tissue samples has also been conducted using the anti-Tn monoclonal IgM BaGs6 (CA3638) which was obtained from the ascites of mice immunized with Tn-expressing cells. BaGs6 has been described as recognizing glycoconjugates containing GalNAcα1-*O*-Ser/Thr but not blood group A or similar glycans terminating in GalNAc, though its formation is experimentally variable due to the nature of the use of unmodified mouse ascites [19, 20]. The Tn antigen has been detected using this reagent in over 90% of CRC, seldom in normal colorectal tissue, and to some degree in histologically normal-appearing peritumoral mucosa, though too few peritumoral samples have been examined to establish definitive Tn expression patterns [18].

We generated a recombinant antibody derived from BaGs6 and produced it in both a recombinant murine IgM form (ReBaGs6) and a human IgG1 form (Remab6) [21]. ReBaGs6 is recombinant expressed in human HEK293 cells and provides a reagent with remarkably high affinity and specificity for clustered Tn antigen. This has been previously validated using a Tn glycopeptide microarray containing various peptides with one or more Tn antigens, including peptides modeled to mimic the hinge region of human IgA1; while ReBaGs6 demonstrated high affinity for di- and tri-Tn structures on mucin-derived glycopeptides, it only weakly bound to glycopeptides modeled after IgA1 and other non-Tn terminal GalNAc structures such as blood group A glycans [21]. Additionally, ReBaGs6 has demonstrated efficacy in immunohistochemical applications. While binding of ReBaGs6 was not observed in normal murine intestinal tissues, extensive staining was observed in tissues from mice engineered to express Tn antigen via intraepithelial knockout of *COSMC (C1GalT1C1)*, which encodes an essential chaperone required for elaboration of O-glycans under physiologic circumstances [21].

The objective of this study is therefore to provide the first detailed immunohistochemical analysis of Tn and STn expression patterns in CRC, and in matched benign peri-tumoral colonic mucosa, and mucosa distant from the tumor, using these well-defined monoclonal antibodies, with comparison to VVA lectin staining.

## Materials and methods

### Human specimens

**Fig. 1:**
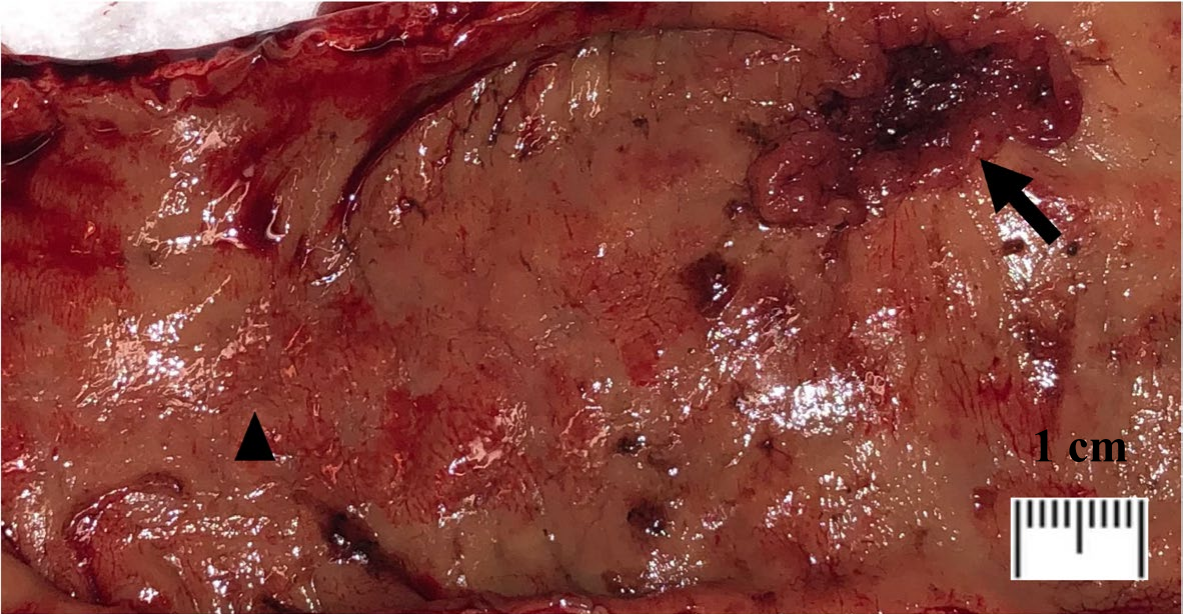
Surgical specimen, gross; upper rectum, rectal adenocarcinoma (arrow), distal normal mucosa (arrowhead)

Human tissue samples were obtained both from 12 prospectively enrolled patients and retrospectively from the archived specimens of 36 patients. For prospectively obtained tissue samples, harvest was conducted using freshly obtained colorectal resection specimens from patients undergoing surgical resection at BIDMC (example shown in Fig. 1). For the 9 patients with CRC, samples were taken from representative portions of the tumor (1–3 samples), normal appearing mucosa at the edge of the tumor (peritumoral), and normal mucosa distant from the tumor (> 3 cm away). All samples were immediately placed in 20% formaldehyde solution and underwent paraffin-embedding and processing by the institutional histology core.

For retrospective analysis, archival paraffin blocks were obtained from the Department of Anatomic Pathology (Beth Israel Deaconess Medical Center, Boston) and for each case, samples from both the gross CRC lesion itself and its matched distant normal tissue were analyzed.

Additionally, a total of 21 adenomatous colorectal tissue samples and matched distant normal mucosal samples, were used (Supplementary tables 1b, 2b, and 3b). Among these, 14 were obtained from archived specimens, and 7 from prospectively obtained specimens. Among those specimens obtained prospectively, 4 featured adenomatous mucosae adjacent to CRC while 3 had adenomatous polyps in absence of malignancy. In total, we were able to grossly obtain transitional margin tissue from 41 specimens. However, there was not always enough tissue of histologic quality/cellularity to be able to stain and score the corresponding slides adequately. Therefore, Supplementary table 2a shows the results of TAG staining for 41 samples, while Supplementary table 1a shows results of ReBaGs6 antibody staining for only 39 samples.

A subset of specimens was sampled randomly for control IHC staining with polyclonal mouse isotype IgM, IgG or VVA (Supplementary table 3a) pre-incubated with free N-Acetyl-D-galactosamine (GalNAc), as appropriate.

The presence of adenocarcinoma, adenoma, and normal mucosa was confirmed by examination of H&E stained sections. For all specimens, the transitional margin (TM) mucosa was defined as histologically benign non-dysplastic colorectal mucosa located within 1 mm away of neoplastic cells. All grading and staging was based on the World Health Organization (WHO) classification of tumors of the colon and rectum 5^th^ edition (2019) [22]. TM tissue was available for analysis in the tissue samples of all of the 9 patients enrolled prospectively and in 30 (86%) of the retrospective archival 35 CRC cases. Among archived cases, CRC lesion tissue was unavailable for 1 case, and matched distant normal tissue was unavailable for 1 case. Usage of all specimens was reviewed and approved by the BIDMC Human Subjects Institutional Review Board (IRB) with informed consent from patients where appropriate and samples were de-identified.

### Immunohistochemistry (IHC)

Tissue sections were deparaffinized, rehydrated, and washed with water. Antigen retrieval was done by heating slides in a pressure cooker for 3 min in citrate buffer (10 mM trisodium citrate, pH 6.0). After cooling down at room temperature, tissue sections were incubated with 3% hydrogen peroxide (Cat#216,763, Sigma) and then blocked with 10% goat serum (Cat#16,210,064, Gibco) or 10% bovine serum albumin (Cat#BP1600-1, Fraction V, Fisher Scientific) in Tris-buffered saline with 0.1% Tween-20 (TBST). Tissue sections were then incubated with primary antibodies at 4 °C overnight, followed by HRP-conjugated goat anti-mouse IgM (Cat#115–035-075, Jackson ImmunoResearch), or anti-mouse IgG (Cat#115–035-062, Jackson ImmunoResearch) secondary antibodies at 1:200 dilution in TBST at room temperature for 1 h. Primary antibodies used in this study included those against Tn antigen (ReBaGs6, mouse IgM [2 µg/mL] [21] and STn antigen (TAG-72, mouse IgG [1 µg/mL], Cat#sc-20042, Santa Cruz). Additionally, biotinylated *Vicia villosa* Lectin [2 µg/mL] (Cat#B-1235, Vector Laboratories) was used for primary detection, and HRP-conjugated streptavidin (Cat#SA-5014, Vector Laboratories diluted at 1:200 in TBST) was applied as secondary reagent. Negative control staining in a randomly selected subset of specimens was carried out using polyclonal isotype mouse IgM [2 µg/mL] (Cat# M31507, Thermo Fisher Scientific), polyclonal isotype mouse IgG [1 µg/mL] (Cat#1033–05, Southern Biotech), and VVA pre-incubated with free N-Acetyl-D-galactosamine (GalNAc) monosaccharide (Cat#MA04390, Biosynth Carbosynth Ltd., diluted to 100 mM at final concentration), respectively. Signals were visualized by incubating sections with Aminoethylcarbazole (AEC) substrate solution (Cat#001,122, Invitrogen), and cell nuclei were counterstained with hematoxylin (Cat#MHS32, Sigma). Whole tissue sections were mounted in CLEAR-MOUNT solution (Cat#17,985–15, Electron Microscopy Sciences) and reviewed by microscopy. ﻿Slide images were analyzed and photographed using SlideAtlas, a high-performance web-based client–server system for digital pathology.

### Immunohistochemical staining evaluation

Each cell population (invasive adenocarcinoma (CRC); transitional mucosa adjacent to carcinoma; adenomatous mucosa; normal mucosa distant from tumor) was scored independently. Intracellular staining intensity was graded from 0 (no staining) to 3 + (intense staining) following which a Histo-score (H score) ranging from 0 to 300 was calculated for each cell type using the following formula: [0 × (% cells 0) + 1 × (% cells 1 +) + 2 × (% cells 2 +) + 3 × (% cells 3 +)] [23].

### Statistical analysis

Paired sample Wilcoxon rank sum tests were used to compare staining H scores between cell type categories; CRC cells, TM cells, and distant normal cells were compared, as were adenoma cells and distant normal cells, respectively. All tests were two-sided, and *p*-values less than 0.05 were considered statistically significant.

## Results

Tissue samples from a total of 45 CRC patients were analyzed, including 9 from prospectively enrolled patients and 36 retrospectively from archived specimens. Among these, samples from 18 (38%) patients also contained adenomatous tissue. Prospectively collected tissue samples from an additional 3 patients with adenomas were also analyzed. Demographic and clinicopathologic data are presented in Table 1.

**Table 1:** Clinicopathologic Data

	**CRC** *n* = 45	**Adenoma** *n* = 21
Enrollment		
Retrospective	36 (80)	14 (67)
Prospective	9 (20)	7 (33)
Age at surgery (yrs)	60 (54–70)	63 (55–72)
Gender	23 Male (51)	12 Male (57)
	22 Female (49)	9 Female (43)
Tumor site		
Colon	35 (78)	17 (80)
Rectum	10 (22)	4 (20)
Gross tumor size (cm)	3 (2.6–5.5)	2.8 (1.7–5.5)
Histopathologic Features		
Well differentiated/low grade	38 (84)	16 (76)
Poorly differentiated/high grade	4 (9)	5 (24)
Mixed	2 (4)	-
Staging		
T Stage		
T0	0	21 (100)
T1	8 (18)	
T2	7 (16)	
T3	23 (51)	
T4	7 (16)	
N Stage		-
N0	25 (56)	
N1	10 (22)	
N2	10 (22)	
M Stage		-
M0	38 (80)	
M1a	7 (20)	
M1b/1c	0	
Neoadjuvant therapy	10 (22)	-

Data presented as n (%) or median (interquartile range)

### Tn antigen expression

**Fig. 2:**
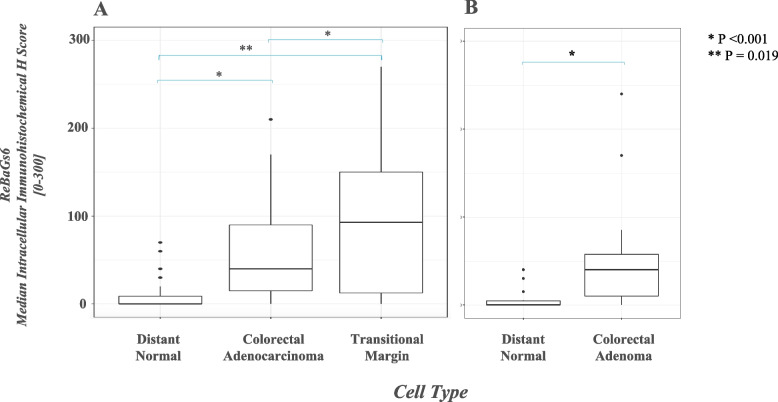
Box plot diagram showing median intracellular immunohistochemical H scores [0–300] in ReBaGs6-stained specimens among patients with CRC, by cell type (**A**). Box plot diagram showing median intracellular immunohistochemical H scores [0–300] in ReBaGs6-stained specimens among patients with adenoma, by cell type (**B**)

**Fig. 3:**
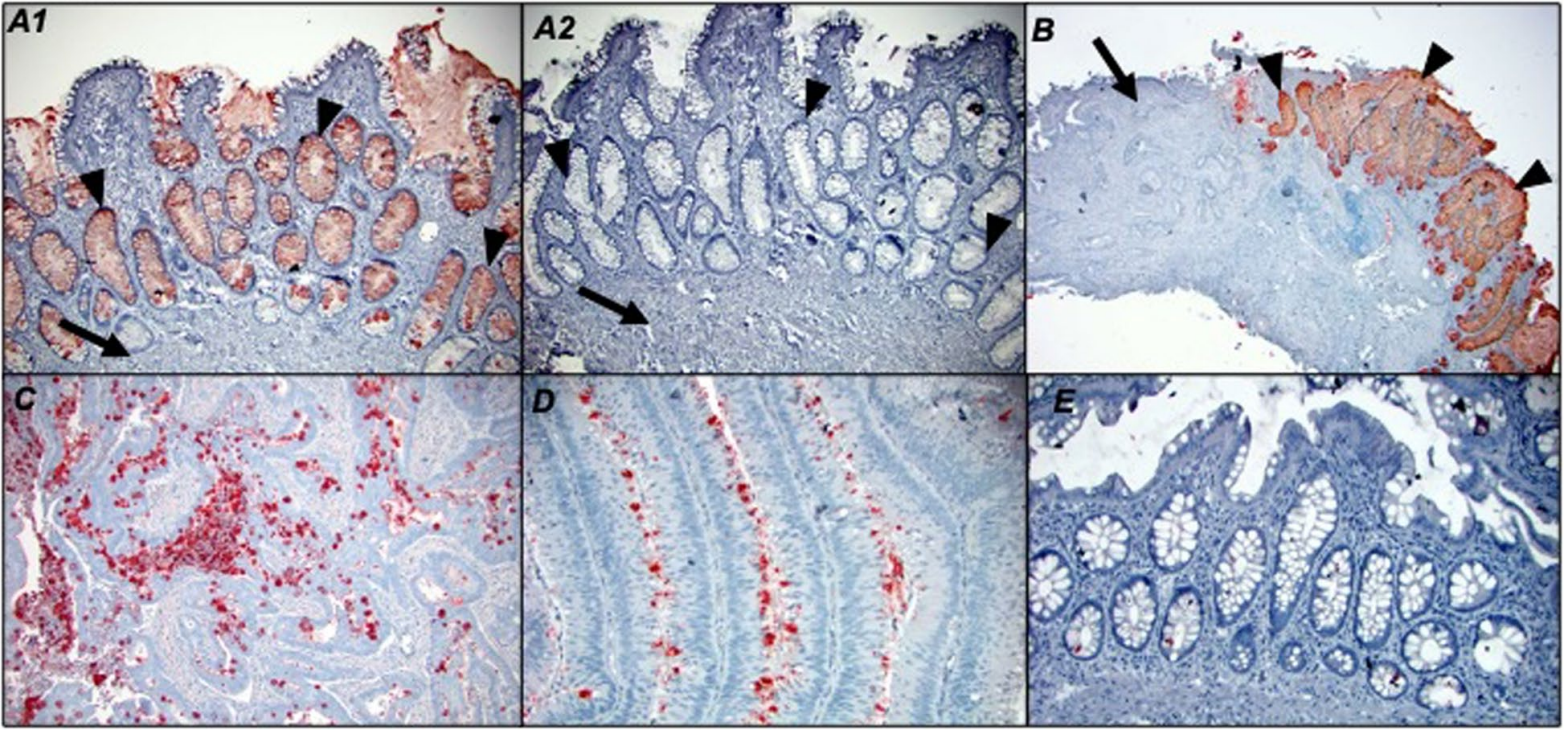
Colon adenocarcinoma (arrows), transitional margin (arrowheads) at 100 × magnification treated with ReBaGs6 at 2 μg/mL and haematoxylin (**A**1) and control isotype mouse IgM and haematoxylin (**A**2); Colon adenocarcinoma and transitional margin at 40 × magnification treated with ReBaGs6 at 2 μg/mL and haematoxylin (**B**); Colon adenocarcinoma at 100 × magnification treated with ReBaGs6 at 2 μg/mL and haematoxylin (**C**); Colon adenoma at 200 × magnification treated with ReBaGs6 at 2 μg/mL and haematoxylin (**D**); Normal distant colon, 3 cm away from adenocarcinoma (not shown), at 200 × magnification, treated with ReBaGs6 at 2 μg/mL and haematoxylin (**E**)

In colorectal adenocarcinoma patients, the majority of tumors (42/43) were positive for Tn antigen as assessed by ReBaGs6 staining (median intracellular H score of 40, range 0–210), and 32/39 foci of mucosa in the transitional margin were also positive (median H score 92.5, range 0–270) (Fig. 2A). Intracellular Tn expression was significantly higher in both TM mucosa (*p* < 0.001) and adenocarcinoma (*p* < 0.001) (Fig. 3-A1, B, C, D), compared to distant normal mucosa (16/41 patients positive, median H score 0, range 0–70). (Fig. 3-E). Furthermore, TM mucosa demonstrates a Tn expression level significantly elevated relative to adenocarcinoma (*p* = 0.019). A representative tissue staining with ReBaGs6 and hematoxylin is presented, highlighting the distinct staining of TM areas (Fig. 3-A1, B). In contrast, the staining level in adenomatous epithelium (18/20 cases positive; median intracellular H score 40; range 0–240) was also higher than that of paired normal tissue (Fig. 3-D, Fig. 2B). Immunostaining using negative control antibodies in a randomly selected subset of specimens yielded negligible staining (Fig. 3-A2).

### STn antigen expression

**Fig. 4:**
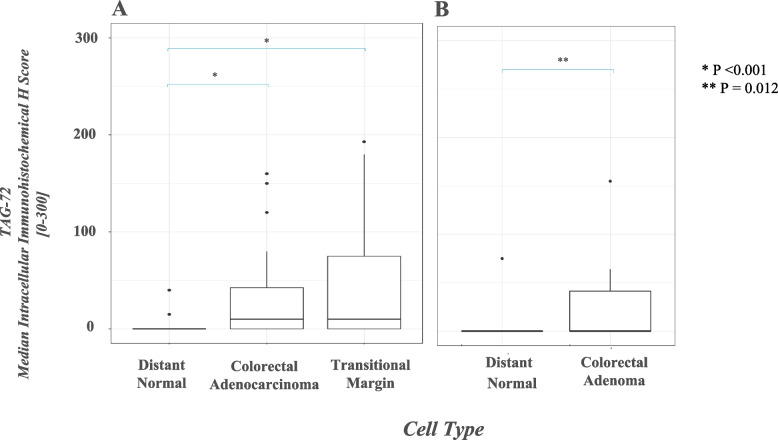
Box plot diagram showing median intracellular immunohistochemical H scores [0–300] in TAG72-stained specimens among patients with CRC, by cell type (**A**). Box plot diagram showing median intracellular immunohistochemical H scores [0–300] in TAG72-stained specimens among patients with adenoma, by cell type (**B**)

**Fig. 5:**
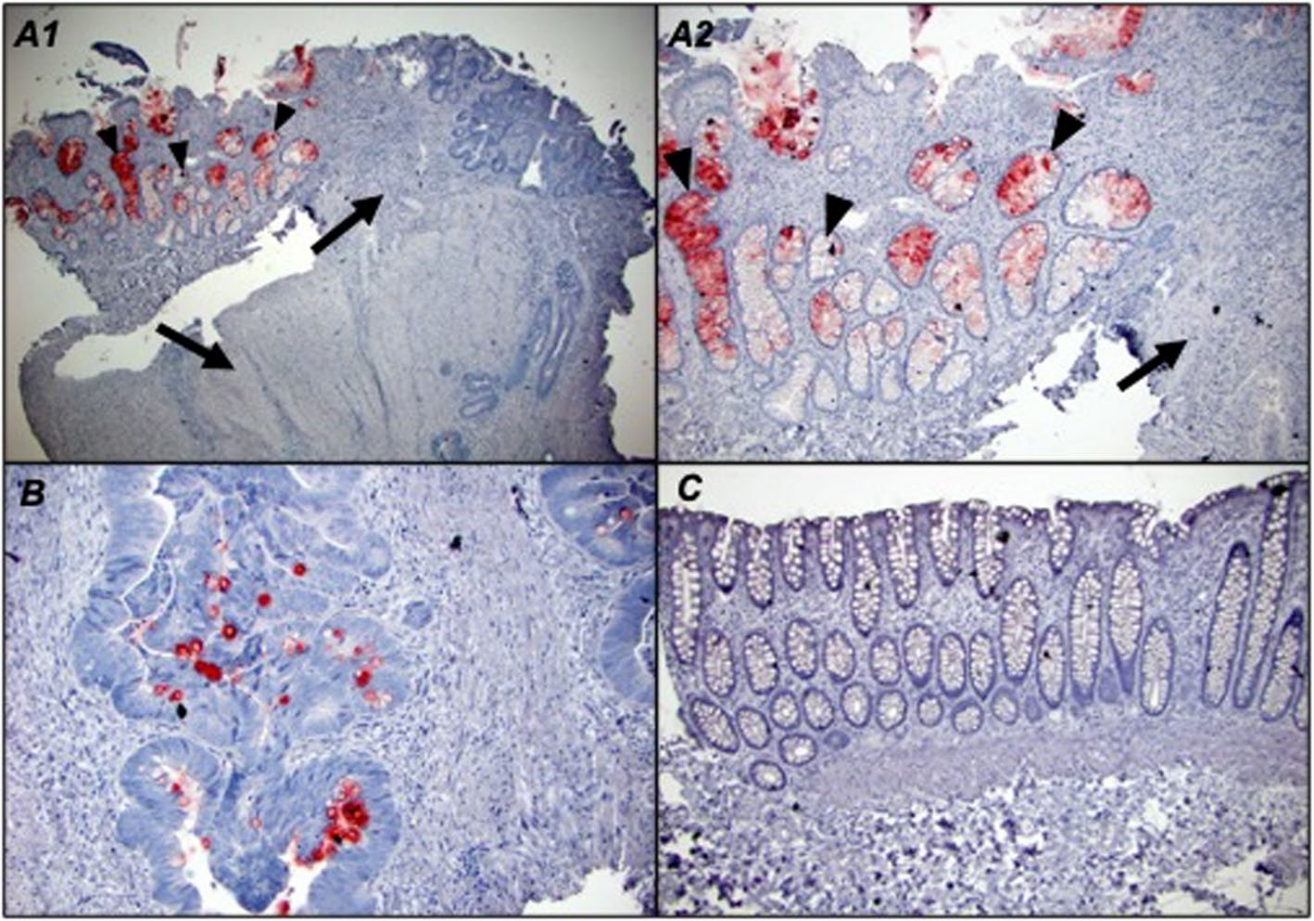
TAG-72 at 1 μg/mL and haematoxylin –treated: colon adenocarcinoma (arrows) and transitional margin (arrowheads) at 40 × magnification (**A**1); and 100 × magnification (**A**2); colon adenocarcinoma at 100 × magnification (**B**); and normal distant colon, 3 cm away from adenocarcinoma (not shown), at 100 × magnification (**C**)

Expression of the STn antigen was assessed using TAG-72, murine IgG1 hybridoma-derived antibody (B72.3) [24]. Staining for STn antigen was present in 30/43 adenocarcinomas (median intracellular H score 10, range 0–160), in of 27/41 foci of TM mucosa (median intracellular H score 10, range 0–192). (Fig. 4A). The STn expression level in TM mucosa not significantly different from that of adenocarcinoma (*p* = 0.11). In contrast, TAG-72 staining was observed in only 2 of 43 examples of distant normal mucosa (median score 0, range 0–40) (Fig. 5-C), a level significantly lower than both TM mucosa (*p* < 0.001) and adenocarcinoma (*p* < 0.001) (Fig. 5-A1, A2, B). Analysis of TAG-72 staining was found in 8/19 adenomas the median intracellular H score in adenomatous cells of 0 (range 0–155) (Fig. 4B). As noted before, the normal mucosa was largely negative for TAG-72 (1/19 cases positive, median H score 0, mean H score 4, range 0). A subset of specimens was stained with negative control antibodies and these yielded negligible staining.

### Terminal GalNAc expression

**Fig. 6:**
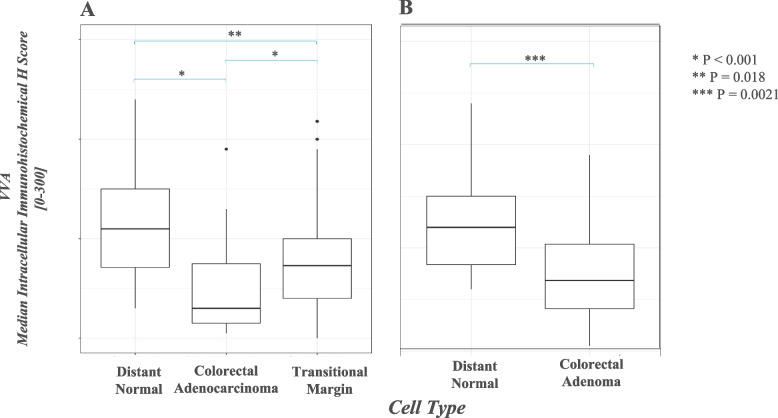
Box plot diagram showing median intracellular immunohistochemical H scores [0–300] in VVA-stained specimens among patients with CRC, by cell type (**A**). Box plot diagram showing median intracellular immunohistochemical H scores [0–300] in VVA-stained specimens among patients with adenoma, by cell type (**B**)

**Fig. 7:**
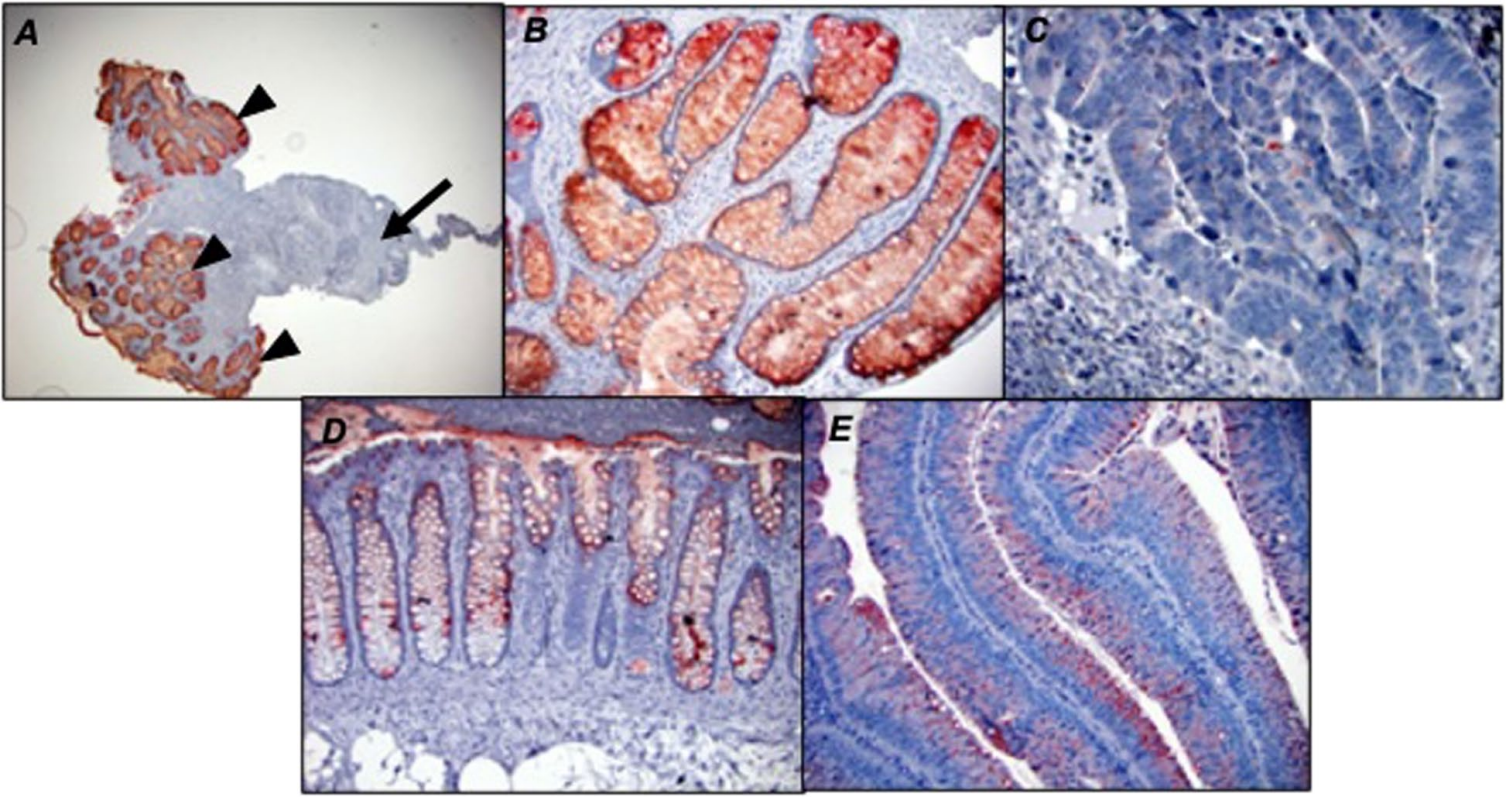
VVA lectin at 2 μg/mL and haematoxylin –treated: colon adenocarcinoma (arrows) and transitional margin (arrowheads) at 40 × magnification (**A**); transitional margin at 100 × magnification (**B**); colon adenocarcinoma at 200 × magnification (**C**); normal distant colon, 3 cm away from adenocarcinoma (not shown), at 100 × magnification (**D**); colon adenoma at 100 × magnification (**E**)

All adenocarcinomas were positive for staining with VVA, a marker of terminal GalNAc expression, as also may occur on blood group A antigens (median H score 30, range 5–190), as were nearly all foci of TM mucosa (41/42 positive, median H score 72.5, range 0–218), and all examples of distant normal mucosa (median H score 110, range 30–240) (Fig. 6A). The level of staining in normal mucosa (Fig. 7-D) was significantly greater than both TM mucosa (Fig. 7-A, B) (*p* = 0.018) and adenocarcinoma (Fig. 7-C) (*p* < 0.001). TM mucosa also demonstrated significantly elevated staining relative to adenocarcinoma (*p* < 0.001). Analysis of VVA staining in adenomas demonstrated a median intracellular H score of 68.5 (range 5–190) in adenomatous cells (Fig. 7-E), which was significantly lower than that of paired normal tissue (median H score 120, range 60–240, *p* = 0.0013) (Fig. 6B). A subset of specimens was sampled randomly to undergo negative control IHC staining, and these yielded negligible staining.

## Discussion

Our study characterizes Tn and STn expression patterns in colorectal adenocarcinomas and adenomas, as compared to matched benign peritumoral and distant normal mucosal tissue sampled from the same patients. Minimal intracellular Tn staining was observed in normal mucosa, while higher Tn expression was observed in both TM mucosa and in adenocarcinoma, a pattern that to a lesser degree was reflected by STn expression in these tissue types. Notably, TM mucosa demonstrates a Tn expression level significantly higher than that of the adenocarcinoma itself. Colorectal adenomas demonstrated greater Tn and STn expression relative to normal mucosa. Tn and STn antigens were seldom observed in normal mucosa.

The histologic evolution of CRC is complex, as is the resulting architecture of the tumor; while a majority of tumors arise in the background of pre-existing benign adenomatous change, others arise de novo [25]. The morphology of tumors is similarly variable, ranging from vegetative to flat and infiltrative, and gross tumor specimen frequently contains regions of histologically normal-appearing or adenomatous tissue adjacent to malignant cells [25]. The presence of multiple cell types in a given specimen can complicate the interpretation of immunohistochemical studies and underscores the value of detailed histopathologic analysis and correlation, which is lacking in much of the current literature [23]. As a result, much of the current characterization of Tn antigen expression in colorectal tissue relies on descriptive or qualitative scoring [23, 26]. In this study, we have utilized a systematic semi-quantitative approach to provide a more detailed histopathologic analysis of intracellular Tn antigen expression patterns by cell type.

In addition to formal histopathologic analysis, this study utilized ReBaGs6, a novel recombinant anti-Tn monoclonal murine IgM specific to clustered Tn antigen but which unlike VVA does not bind to other terminal α-linked GalNAc residues, such as blood group A glycans, to provide the first detailed immunohistochemical analysis of Tn expression in matched samples of adenocarcinoma, peritumoral transitional margin, and normal mucosa from the same patient [21]. The expression of the Tn antigen has historically been evaluated using other monoclonal antibodies; however, these monoclonal antibodies may cross-react with terminal GalNAc residues on normal glycans, including blood group A, and have not been well-defined in specificity in terms of glycopeptide recognition and the independent recognition only of the glycan antigen. Similarly, plant lectins may recognize terminal GalNAc structures including blood group A, Forssman-related antigens, and IgA1 in human circulation [27]. In contrast, ReBaGs6 is an antibody specific for the Tn antigen which does not cross-react with any other GalNAc-related structures and has no recognition of peptide elements. This antibody has been well-characterized, to a further extent than other available antibodies, and is more specific to Tn compared to VVA or other lectins that bind more broadly [21].

Immunohistochemistry for ReBaGs6 revealed Tn expression patterns in CRC that are to some degree similar to prior studies, which had demonstrated relatively high prevalence of Tn antigen in CRC using lectins and less well-defined antibodies [16, 18]. Novel in our study, however, is the discovery of elevated levels of expression of Tn and STn antigens in benign peritumoral transitional margin (TM) mucosa, when compared to normal colonic mucosa more distant from the tumor and even to the adenocarcinoma itself. This finding establishes both the TM and adenocarcinomas candidate sources of Tn and STn-bearing glycoproteins, which may ultimately be detectable in the circulation and likely represent valuable potential biomarkers or antibody-based therapy targets [28]. Historically, several potentially Tn-bearing glycoproteins, including mucins CA15-3 (MUC1) and CA125 (MUC16), have been developed for clinical use in following cancer progression; however, the expression and identification of mucin protein epitopes are not specific enough for use in cancer screening [29–31]. To date the authors are unaware of any current publications evaluating expression of Tn or STn antigens in systemic circulation, and this represents a productive goal for future study in our lab, along with evaluating the different clinical factors that can impact Tn & STn expression and are routine use in the management of colorectal cancer, such as a neoadjuvant chemotherapy and radiotherapy, which have not been studied to date.

Some expression of Tn and STn was also seen in precursor adenomatous epithelium. Prior studies have also described increased expression of Tn and STn in these lesions, though the degrees of expression varied widely, from 50 to over 90%, and normal control tissue from the same patient was often not examined [32–35]. Our findings may have important mechanistic implications with regard to the relationship of O-glycosylation aberrancies and malignant transformation in CRC; just as microenvironmental carcinogenic exposure promotes progression along the adenoma-carcinoma sequence, similar extrinsic factors may account for the acquired impairment in physiologic O-glycosylation pathways once an adenoma is already present [36]. It is also likely that the microenvironmental milieu which promotes malignant transformation to CRC also promotes evasion of host immunity and thus confers metastatic potential; this suggests that the impairment of typical O-glycosylation pathways observed in transitional margin mucosa may result from the localized microenvironmental impact of CRC [37].

In this study, the detection of Tn and STn expression patterns in CRC and premalignant tissue was in large part made possible by the detailed cell-type specific histopathologic analysis used in this study, rather than estimation based on gross specimen classifications as has been done in prior studies [18]. Our results also show that patterns of Tn expression in CRC and TM are largely reflected by similar trends in STn antigen expression, as has been described in existing literature [18, 38], though notably the levels of STn expression in CRC and TM tissue were not significantly different.

There are several notable limitations of this study. Among these are the relatively small sample size as well as the semi-quantitative nature of immunohistochemical stain evaluation. In addition, while specimens were sampled systematically and at multiple locations including within the tumors, the possibility of sampling error cannot be excluded.

The elucidation of these patterns is of potentially great clinical significance as it raises important mechanistic questions. While early alteration of Tn expression in preneoplastic epithelium may play a requisite role in malignant transformation, it is also possible that Tn expression changes result from the microenvironmental or paracrine effects of tumor on TM mucosa. Future expansion of this analysis may ultimately offer candidate Tn-positive glycoproteins that are unique to early cancer lesions and adenomatous precursor lesions.

The results of this study are based upon the use of the recombinant anti-Tn monoclonal murine IgM ReBaGs6, which is highly specific for clustered Tn structures and which has been well-characterized and sequenced [21], making it accessible and highly advantageous relative to less specific lectins or lab-engineered hybridomas that may be laborious to consistently reproduce.

## Supplementary Information

**Additional file 1: Supplementary Table 1a.** Results of immunohistochemical staining with ReBaGs6 (2 µg/mL) in colorectal cancer specimens. **Supplementary Table 1b.** Results of immunohistochemical staining with ReBaGs6 (2 µg/mL) in colorectal adenoma specimens. **Supplementary Table 2a.** Results of immunohistochemical staining using TAG-72 (1 µ g/mL) in colorectal cancer specimens. **Supplementary Table 2b.** Results of immunohistochemical staining using TAG-72 (1 µg/mL) in colorectal adenoma specimens. **Supplementary Table 3a.** Results of immunohistochemical staining using VVA lectin (2 µg/mL) in colorectal cancer specimens. **Supplementary Table 3b.** Results of immunohistochemical staining using VVA lectin (2 µ g/mL) in colorectal adenoma specimens.

## Data Availability

The authors confirm that the data supporting the findings of this study are available within the article and its supplementary materials.
