# Supplementary material for: Immunohistochemical analysis of Tn antigen expression in colorectal adenocarcinoma and precursor lesions
Source: BMC Cancer. 2022 Dec 7;22:1281. doi: 10.1186/s12885-022-10376-y (PMC9730631; doi:10.1186/s12885-022-10376-y)
Supplement: Supplementary file 1 — Additional file 1: Supplementary Table 1a. Results of immunohistochemical staining with ReBaGs6 (2 µg/mL) in colorectal cancer specimens. Supplementary Table 1b. Results of immunohistochemical staining with ReBaGs6 (2 µg/mL) in colorectal adenoma specimens. Supplementary Table 2a. Results of immunohistochemical staining using TAG-72 (1 µ g/mL) in colorectal cancer specimens. Supplementary Table 2b. Results of immunohistochemical staining using TAG-72 (1 µg/mL) in colorectal adenoma specimens. Supplementary Table 3a. Results of immunohistochemical staining using VVA lectin (2 µg/mL) in colorectal cancer specimens. Supplementary Table 3b. Results of immunohistochemical staining using VVA lectin (2 µ g/mL) in colorectal adenoma specimens. [file 12885_2022_10376_MOESM1_ESM.docx]

**Supplementary Tables:**

**Supplementary Table 1a**. Results of immunohistochemical staining with ReBaGs6 (2 μg/mL) in colorectal cancer specimens.

| Intracellular staining with ReBaGs6 (2 μg/mL) (H score) | | |
| --- | --- | --- |
| Colorectal Cancer (CRC)  n = 43 | Transitional Margin (TM)  n = 39 | Distant Normal  n = 43 |
| 50 | 130 | 0 |
| 210 | 160 | 10 |
| 130 | 0 | - |
| 60 | 240 | 15 |
| 51 | 30 | 0 |
| 5 | 0 | 0 |
| 55 | 0 | 0 |
| 80 | 45 | 0 |
| 15 | 90 | 0 |
| 120 | - | 0 |
| 120 | 5 | 0 |
| 30 | - | 20 |
| 80 | - | 4 |
| 10 | 110 | 0 |
| 120 | 5 | 0 |
| 40 | 100 | 0 |
| 15 | 60 | 0 |
| 45 | 140 | 4 |
| - | 0 | 5 |
| 100 | 190 | 30 |
| 110 | 60 | 20 |
| 25 | 185 | 30 |
| 120 | - | 0 |
| 15 | 60 | 0 |
| 15 | 0 | 0 |
| 40 | 120 | - |
| 170 | 270 | 40 |
| 0 | - | 0 |
| 35 | 5 | 0 |
| 15 | 20 | 0 |
| 60 | 70 | 5 |
| 5 | 0 | 0 |
| 45 | 140 | 10 |
| 165 | 30 | 0 |
| 55 | 0 | 0 |
| 5 | 170 | 0 |
| 15 | 183 | 0 |
| 20 | 165 | 0 |
| 22 | 93 | 0 |
| 20 | 187 | 0 |
| 13 | 130 | 5 |
| 165 | 135 | 70 |
| 30 | 180 | 40 |
| 5 | 93 | 60 |

**Supplementary Table 1b**. Results of immunohistochemical staining with ReBaGs6 (2 μg/mL) in colorectal adenoma specimens.

| Intracellular staining with  ReBaGs6 (2 μg/mL) (H score) | |
| --- | --- |
| Adenoma  n = 20 | Distant Normal  n = 20 |
| 40 | 0 |
| 82 | 0 |
| 50 | 0 |
| 25 | 0 |
| 55 | 0 |
| 65 | 5 |
| 55 | 40 |
| 240 | 4 |
| 5 | 15 |
| 5 | 0 |
| 0 | 0 |
| 45 | 0 |
| 20 | 0 |
| 20 | 0 |
| 10 | 0 |
| 40 | 30 |
| 170 | 0 |
| 10 | - |
| 85 | 40 |
| 0 | 0 |

**Supplementary Table 2a**. Results of immunohistochemical staining using TAG-72 (1 μg/mL) in colorectal cancer specimens.

| Intracellular staining with TAG-72 (1 μg/mL) (H score) | | |
| --- | --- | --- |
| Colorectal Cancer (CRC)  n = 43 | Transitional Margin (TM)  n = 41 | Distant Normal  n = 42 |
| 5 | 0 | 0 |
| 160 | 100 | 0 |
| 0 | 0 | - |
| 25 | 50 | 0 |
| 60 | 10 | 0 |
| 0 | 5 | 0 |
| 20 | 0 | 0 |
| 20 | 90 | 0 |
| 5 | 0 | 0 |
| 80 | - | 0 |
| 10 | 0 | 0 |
| 10 | 0 | 0 |
| 0 | 0 | 0 |
| 0 | 180 | 0 |
| 50 | 0 | 0 |
| 10 | 10 | 0 |
| 45 | 50 | 0 |
| 15 | 40 | 0 |
| 55 | 90 | 15 |
| 55 | 20 | 0 |
| 5 | 70 | 0 |
| 150 | - | 0 |
| 0 | 80 | 0 |
| 40 | 0 | 0 |
| 10 | 0 | 0 |
| 120 | 10 | 0 |
| 0 | 10 | 0 |
| 10 | 5 | 0 |
| 5 | 0 | 0 |
| 10 | 20 | 0 |
| 0 | 0 | 0 |
| 40 | 120 | 0 |
| 80 | 5 | 0 |
| 55 | 0 | 0 |
| 0 | 75 | 0 |
| 0 | 80 | 0 |
| 12 | 98 | 0 |
| 3 | 19 | 0 |
| 3 | 69 | 0 |
| 0 | 70 | 0 |
| 0 | 0 | 0 |
| 0 | 193 | 0 |
| 0 | 90 | 40 |

**Supplementary Table 2b**. Results of immunohistochemical staining using TAG-72 (1 μg/mL) in colorectal adenoma specimens.

| Intracellular H Scores | |
| --- | --- |
| Adenoma  n = 19 | Distant Normal  n = 19 |
| 0 | 0 |
| 45 | 0 |
| 0 | 0 |
| 5 | 0 |
| 10 | 0 |
| 50 | 0 |
| 0 | 0 |
| 0 | 0 |
| 0 | 0 |
| 30 | 0 |
| 0 | 0 |
| 0 | 0 |
| 0 | 0 |
| 155 | 0 |
| 0 | 0 |
| 64 | 0 |
| 0 | 0 |
| 60 | 0 |

**Supplementary Table 3a.** Results of immunohistochemical staining using VVA lectin (2 μg/mL) in colorectal cancer specimens.

| Intracellular staining with VVA lectin (2 μg/mL) (H score) | | |
| --- | --- | --- |
| Colorectal Cancer (CRC)  n = 43 | Transitional Margin (TM)  n = 42 | Distant Normal  n = 43 |
| 25 | 10 | 180 |
| 190 | 200 | 240 |
| 20 | 20 | - |
| 35 | 140 | 140 |
| 35 | 110 | 120 |
| 5 | 190 | 170 |
| 95 | 80 | 140 |
| 90 | 165 | 80 |
| 65 | 120 | 240 |
| 80 | - | 150 |
| 50 | 15 | 80 |
| 20 | 30 | 110 |
| 10 | 40 | 90 |
| 10 | 80 | 150 |
| 80 | 80 | 120 |
| 80 | 90 | 105 |
| 65 | 60 | 40 |
| 40 | 95 | 75 |
| 105 | 70 | 70 |
| 85 | 70 | 155 |
| 20 | 60 | 70 |
| 130 | - | 165 |
| 30 | 90 | 170 |
| 60 | 130 | 110 |
| 10 | 25 | 70 |
| 60 | 100 | 100 |
| 40 | 65 | 55 |
| 20 | 85 | 135 |
| 85 | 40 | 55 |
| 10 | 55 | 150 |
| 5 | 60 | 110 |
| 25 | 35 | 185 |
| 70 | 115 | 85 |
| 20 | 0 | 65 |
| 15 | 73 | 30 |
| 15 | 47 | 100 |
| 8 | 85 | 160 |
| 22 | 218 | 90 |
| 7 | 113 | 150 |
| 8 | 45 | 175 |
| 128 | 17 | 45 |
| 19 | 100 | 60 |
| 10 | 37 | 55 |

**Supplementary Table 3b**. Results of immunohistochemical staining using VVA lectin (2 μg/mL) in colorectal adenoma specimens.

| Intracellular staining with  VVA lectin (2 μg/mL) (H score) | |
| --- | --- |
| Adenoma  n = 18 | Distant Normal  n = 18 |
| 25 | - |
| 107 | - |
| 25 | 75 |
| 62 | 150 |
| 100 | 175 |
| 25 | 60 |
| 75 | 140 |
| 60 | 120 |
| 190 | 240 |
| 60 | 150 |
| 5 | 80 |
| 150 | 120 |
| 150 | 105 |
| 105 | 165 |
| 60 | 70 |
| 75 | 100 |
| 90 | 135 |
| 35 | 85 |
